# Consequences of Hybridization in Mammals: A Systematic Review

**DOI:** 10.3390/genes13010050

**Published:** 2021-12-24

**Authors:** Roya Adavoudi, Małgorzata Pilot

**Affiliations:** Museum and Institute of Zoology, Polish Academy of Sciences, ul. Nadwiślańska 108, 80-680 Gdańsk, Poland; radavoudi@miiz.waw.pl

**Keywords:** adaptive introgression, genetic swamping, hybridization, hybrid speciation, hybrid zones, outbreeding depression, mammals

## Abstract

Hybridization, defined as breeding between two distinct taxonomic units, can have an important effect on the evolutionary patterns in cross-breeding taxa. Although interspecific hybridization has frequently been considered as a maladaptive process, which threatens species genetic integrity and survival via genetic swamping and outbreeding depression, in some cases hybridization can introduce novel adaptive variation and increase fitness. Most studies to date focused on documenting hybridization events and analyzing their causes, while relatively little is known about the consequences of hybridization and its impact on the parental species. To address this knowledge gap, we conducted a systematic review of studies on hybridization in mammals published in 2010–2021, and identified 115 relevant studies. Of 13 categories of hybridization consequences described in these studies, the most common negative consequence (21% of studies) was genetic swamping and the most common positive consequence (8%) was the gain of novel adaptive variation. The total frequency of negative consequences (49%) was higher than positive (13%) and neutral (38%) consequences. These frequencies are biased by the detection possibilities of microsatellite loci, the most common genetic markers used in the papers assessed. As negative outcomes are typically easier to demonstrate than positive ones (e.g., extinction vs hybrid speciation), they may be over-represented in publications. Transition towards genomic studies involving both neutral and adaptive variation will provide a better insight into the real impacts of hybridization.

## 1. Introduction

Until recently, hybridization was considered as a rare phenomenon in the animal kingdom and thus its role in animal evolution has been underappreciated [1]. Growing amount of whole genome sequence data produced in recent years made it possible to demonstrate that a broad range of animal species have experienced hybridization events [2]. Although hybridization frequency (i.e., the proportion of individuals interbreeding with another species and producing hybrid offspring) is low in most species [3], it has been estimated that around 25% of plant species and 10% of animal species have been affected by hybridization [4]. Hybridization is most frequent among recently diverged sister species, which are frequently characterized with incompletely developed prezygotic isolation (behavioural and mechanical) and postzygotic isolation (zygote mortality and hybrid inviability and sterility) [5]. Hybridization is now recognized as a widespread phenomenon with significant impact on species evolution [6,7] and potentially serious consequences for species conservation and management [8].

Cross-breeding between species with limited postzygotic isolation can often lead to an intake of genetic variation typical of one species into another species’ gene pool—a process called introgression. Introgressive hybridization can affect creation, maintenance and loss of biodiversity [9]. In some cases, introgression may facilitate species evolutionary responses to environmental changes by promoting rapid acquisition of new adaptive genetic variants [10,11], thus increasing adaptive potential of these species [12,13,14,15]. Moreover, introgressive hybridization can contribute to speciation by creating new genetic variation and functional novelty [6,16]. Introgression of alleles from one species into another one can create novel adaptive combinations of alleles and form a new admixed population, which is genetically distinct from both parental populations [17]. Such population may develop reproductive isolation from the parental populations and thus maintain distinctiveness even in a contact zone [18]. However, speciation through hybridization occurs less frequently in mammals compared to other vertebrates, because reproductive barriers between mammalian species are in general well established [19]. In species with low genetic variation, introgressive hybridization could increase genetic variation and decrease inbreeding, without any signs of outbreeding depression [20,21].

On the other hand, hybridization can be also considered as a potential threat to species survival [22,23,24]. Accumulation of deleterious variation [25], outbreeding depression [26,27,28] and genetic swamping [29,30,31] are among detrimental consequences of hybridization. In extreme cases, severe outbreeding depression and decline in the population growth rate below the replacement rate due to wasted reproductive effort in one or both parental lineages may lead to extinction [32]. High risk of extinction due to hybridization has been reported for rare or endangered species interbreeding with more common relatives [33,34]. However, hybridization was mentioned as a factor contributing to extinction in only 11 species out of 120,369 extinct species assessed [23]. The negative impact of hybridization should not be neglected, but the conservation policies should not be focused on the negative aspects of hybridization only [23]. From a conservation perspective, hybridization outcomes may range from considerable introgression with significant negative impacts, e.g., reduced survival or reproductive success of hybrids, through minimal introgression with negligible impact, to moderate introgression with significant positive impacts, e.g., increased fitness of admixed individuals [35]. Given than hybridization can represent either a threat to species survival or a potential pathway to evolutionary rescue, it is important to examine its impacts case-by-case [35].

Hybridization may be particularly common in widespread, abundant species and in non-indigenous species that were intentionally or unintentionally introduced into a new habitat by humans [32,36]. Hybridization has also been frequently reported for domesticated species and their wild relatives, e.g., wild boar and domestic pig [20,37], gray wolf and domestic dog [38,39,40,41,42,43], wild cat and domestic cat [44,45,46,47]. In such cases hybridization may lead to the introgression of gene variants typical for domestic animals into gene pools of wild species [48,49]. This may have a range of negative consequences, such as the loss of specific adaptations [41] and reduced viability [50]. These negative consequences are particularly pronounced in small, fragmented and isolated populations [51]. Moreover, introgressive hybridization can also affect feral populations of domesticated animals [52,53]. Cross-breeding between individuals originating from captive-bred populations and their wild conspecifics may have similar consequences as that between domesticated and wild populations [54].

In recent decades, several review papers on hybridization have been published. They were focused on specific aspects of hybridization and/or particular taxonomic groups, for example the evolutionary importance of natural hybridization [2], the role of hybridization in extinction [32], hybrid fitness [55], introgression during anthropogenic hybridization [56], mammalian hybrid zones [57], taxonomic problems associated with inter-specific gene flow [58], hybridization in European ungulates [59] and hybridization in New Zealand taxa [60]. Most studies to date focused on documenting hybridization events and analyzing their causes, while relatively little is known about the consequences of hybridization and its impact on the parental species [23]. To address this knowledge gap, we conducted a systematic review of studies on hybridization in mammals and assessed the frequency of different consequences of hybridization reported. In addition, we evaluated the contribution of different mammalian orders and families in published studies on hybridization. We selected mammals as the focal class because of the large number of available studies, resulting in part from the profound role of species from this class in ecosystem functioning [61].

## 2. Materials and Methods

We focused on papers on hybridization in mammals published between 2010 and 2021. The database search for papers published in 2021 was completed on the 3rd of December of that year, so papers published after that date are excluded from the results. For finding relevant papers in the Web of Science, we employed the following string: (“hybridization*” OR “hybridisation*” OR “outbreeding*” OR “outcrossing” OR “admixture*” OR “admixed individual*” OR “hybrid zone” OR “hybrid individual$*” OR “backcrosse$”) AND (“mammal$*” OR “vertebrate$” OR “consequence” OR “implication” OR “Extinction” OR “genetic swamping” OR “adaptive introgression” OR “hybrid speciation” OR “outbreeding depression”) NOT (“protein$” OR “fish$” OR “plant$” OR “invertebrate$” OR “avian reptile$” OR “non avian reptile$” OR “fung$” OR “bird$” OR “Lizard$” OR “penguin$” OR “turtle$” OR “insect$” OR “frog$” OR “butterfl$” OR “homoploid” OR “moth$” OR “salamander$”). With these keywords we found limited numbers of relevant papers (49 papers), we therefore applied different sets of keywords: (“hybridization*” OR “hybridisation*” OR “hybrid$*” OR “outbreeding” OR “outcrossing” OR “admixture*” OR “introgression*” OR “admixed individual *”OR “hybrid zone” OR “hybrid individual$*” OR “backcrosse$”) AND (“mammal$ *”) NOT (“protein$” OR “cell$” OR “fish$” OR “plant$” OR “invertebrate$” OR “avian reptile$” OR “non avian reptile$” OR “fung$” OR “Cell$” OR “bird$” OR “Lizard$” OR “penguin$” OR “turtle$” OR “insect$” OR “frog$” OR “butterfl$” OR “homoploid” OR “moth$” OR “salamander$”). We combined the search results based on these two sets of keywords.

**Figure 1:**
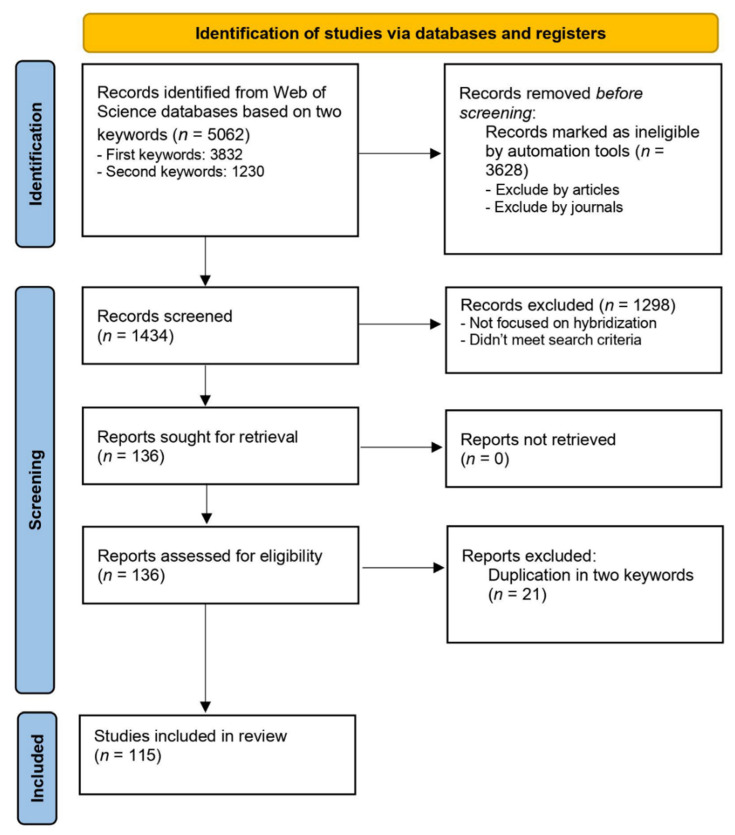
Flow diagram showing the selection stages of studies to be included in the review based on the two sets of keywords used.

We excluded from the search books, review papers, theses, annuals or meeting reports. We also excluded papers published in journals that were outside of the following categories: Evolutionary Biology, Genetics and Heredity, Ecology, Biology, Zoology, Biodiversity Conservation, Multidisciplinary Science and Biochemistry and Molecular Biology. Abstracts of the papers that were identified after applying this automatic exclusion (793 and 641, respectively) were read and these papers that were not focused on the hybridization process in mammals were removed. We also removed one of the two copies of papers that overlapped between the paper sets resulting from the search with each set of keywords (Figure 1). We then combined the results from the two sets of papers, including only the papers meeting the following criteria: experimental studies, focused on mammalian species, that investigate hybridization among different species and subspecies. Studies that evaluated admixture among populations of the same species were removed from the analysis, with the exception of wild and domesticated forms that have been classified as the same species (e.g., wild cat *Felis silvestris silvestris* and domestic cat *Felis silvestris catus*), as well as wild vs captive/farmed populations of the same species. Although we designed the search terms to be as comprehensive as possible in the detection of studies on mammalian hybridization, some relevant studies could have been missed. Nevertheless, the resulting set of papers is free of human bias (except for the choice of keywords) and therefore provides a reliable overview of the current knowledge on the consequences of hybridization.

## 3. Results

According to the inclusion criteria defined above, a total of 49 and 87 published papers were selected based on the first and second set of keywords. This was a small subset of papers initially identified with each set of keywords; we tried multiple versions of keywords and did not manage to improve the accuracy of results. After excluding overlapping papers between the two sets of keywords (15.4%), 115 papers were retained for the subsequent analysis (Figure 1). These papers are listed in Supplementary Table S1.

**Figure 2:**
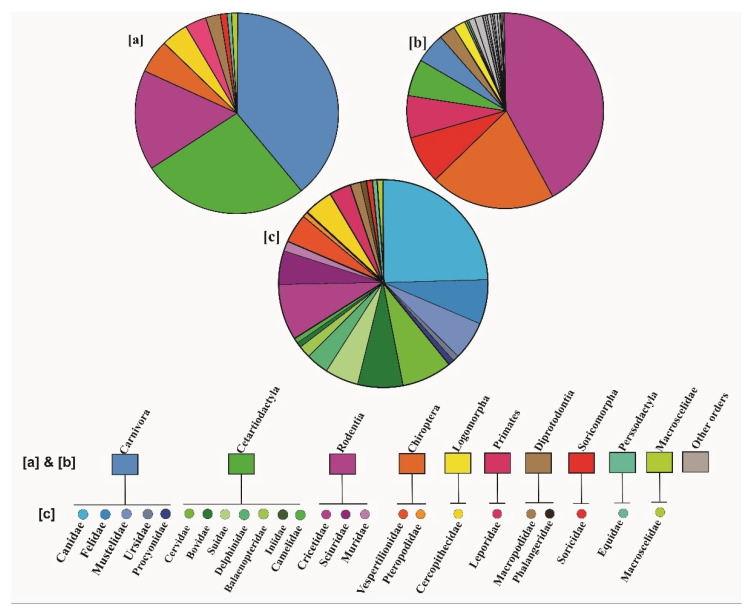
Relative frequencies of (**a**) orders represented in 115 papers included in the systematic review, (**b**) orders represented across all currently recognized mammalian species [62] and (**c**) families represented in 115 papers included in the systematic review.

We calculated the frequency of mammalian orders and families involved in hybridization process in the surveyed studies (Figure 2). 39.13% of these studies focused on the order Carnivora (45 papers), of which Canidae (24.34%, 28 papers), Felidae (6.95%, eight papers), and Mustelidae (6.08%, seven papers) were the most-studied families. Order Cetartiodactyla was the second most-studied order (26.95%, 31 papers), in which Cervidae (7.82%, nine papers), Bovidae (6.95%, eight papers) and Suidae (5.21%, six papers), were the most-studied families. The third most represented order was Rodentia (15.65%, 18 papers), in which Cricetidae (8.69%, 10 papers), Sciuridae (5.21%, six papers) and Muridae (1.75%, two papers) had the largest contribution. Other orders were represented by only two families (Chiroptera, 5.21%, six papers and Diprotodontia 2.60%, three papers) or one family (Primates (3.40%, four papers), Lagomorpha (4.34%, five papers), Macroscelidea (0.86%, one study), Perissodactyla (0.86%, one study) and Soricomorpha (0.86%, one study).

We compared the frequencies of species representing different mammalian orders in the surveyed studies on hybridization with the frequencies of all currently recognized contemporary mammalian species [62] representing different mammalian orders. This comparison demonstrated a considerable bias in the number of studies on hybridization focused on representatives of different mammalian orders (Figure 2a,b).

We classified the hybridization outcomes described in the 115 papers included in the systematic review into 13 categories (Table 1). These categories were non-exclusive and in some cases the consequences of hybridization reported could be classified to more than one category. Among the 115 surveyed papers, 10 papers did not provide sufficient information to classify them in any category, e.g., [63,64]. Therefore, this classification is based on 105 papers. The reported frequencies of different hybridization outcomes in these papers should not be considered as reliable estimates of the real frequencies, because of the biases discussed below. We classified the impacts of each possible outcome as (1) positive (e.g., gaining novel adaptive variation), (2) neutral or unknown, (3) negative (e.g., extinction, loss of reproductive output) and (4) considered as negative. In this last category we included genetic swamping and introgression from a domesticated lineage, which are frequently described as negative in the literature [29,30,65,66,67]. Given that direct empirical evidence for their negative effects is limited, we did not classify them as unequivocally negative outcomes. It is important to note that extinction due to extreme genetic swamping is classified in a separate category, given its clearly negative impact.

Of 115 studies considered, 21 (18.26%) identified hybrids using microsatellite loci as the only genetic markers, 18 studies (15.65%) used mtDNA fragments, 35 (30.43%) studies used both microsatellite loci and mtDNA, 12 studies (10.43%) used genome-wide single nucleotide polymorphisms (SNPs) or whole genome sequencing, two studies (1.73%) used all these three types of markers, and the remaining studies used another method of hybrid detection. Altogether, 50.42% of studies used microsatellite loci as either the only type of genetic markers or together with other types.

To assess whether the analysis of genome-wide data may affect the type of hybridization outcomes observed, we calculated the frequencies of different outcomes based on 14 papers that used genome-wide SNPs or whole genome sequencing. As more than half of these papers (eight studies) focused on hybridization between domestic animals and their wild relatives, introgression from a domesticated lineage was the most common negative effect (36% of studies), followed by genetic swamping (18%). Novel adaptive variation was the only positive impact of hybridization and was reported in five studies (23%). In total, negative outcomes were reported more frequently than positive ones (54% and 23%, respectively). The frequencies of both negative and positive outcomes were higher than in the entire dataset (49% and 13%), while the frequency of neutral outcomes was smaller. However, these frequencies should be treated with caution due to the small number of studies and overrepresentation of hybridization with domestic animals among the studies considered.

## 4. Discussion

### 4.1. Hybridization in Mammalian Orders and Families

#### 4.1.1. Mammalian Orders

The frequency of different mammalian orders in the studies included in this systematic review does not reflect the number of species within each order. Representatives of orders Carnivora and Cetartiodactyla prevail among the species studied, with the frequencies of 39% and 27%, respectively. The frequency of species from these orders among all mammalian species are 5% and 6%, respectively [62]. In contrast, two most species-rich mammalian orders, Rodentia (42% of species) and Chiroptera (21% of species) were represented in only 16% and 5% of studies, respectively. In studies of hybrid zones, rodents have been represented more frequently than other mammalian orders, but nevertheless only eight rodent genera have been studied, as reported in a review paper [57]. Therefore, the underrepresentation of rodents in hybridization studies may result from the focus on well-studied genera only, such as e.g., the genus *Mus*. Several studies published in the previous decade (i.e., not considered in this systematic review) detected signatures of hybridization in several bat species e.g., [68,69,70,71], but altogether hybridization was reported for less than 20 of over 1000 known bat species. This may be associated with a limited number of studies on hybridization in bats [72] or a stronger reproductive isolation in bat species compared with other mammals [70,73]. As bats can form mixed-species groups during mating seasons [74] and during maternal care [75], reproductive barriers are particularly important for the maintenance of species distinctiveness.

Accordingly, overrepresentation of orders Carnivora and Cetartiodactyla in hybridization studies may result from more relaxed reproductive barriers between congeneric species from these orders compared with other mammals, overrepresentation of studies focused on these orders, or a combination of both. High interest in studying species from these orders may result from their important roles in ecosystems and in some cases their high commercial value. Large species from the order Carnivora are keystone species in their ecosystems and are frequently subject to active management and conservation strategies [76,77,78,79]. Accordingly, many representatives of Artiodactyla are valuable game species. Moreover, species from Carnivora and Cetartiodactyla orders can compete with humans over resources by consuming game species, livestock depredation and fisheries depredation, as well as damaging crops and wild vegetation [78,80]. For these reasons, they are of particular interest to wildlife researchers, also in the context of hybridization.

In theory, the proportion of species within each mammalian order that are subject to introgressive hybridization could be used as a measure of the strength of reproductive barriers between species within each order. However, to achieve a reliable comparison between mammalian orders, several sources of bias would have to be accounted for, including the above-mentioned differences in intensity of research on different mammalian orders as well as differences in criteria used to define species. Therefore, in practice the frequency of detected hybridization cases is not a reliable measure of the strength of reproductive isolation.

#### 4.1.2. Mammalian Families

The frequency of families within each mammalian order that are subject to hybridization studies is biased as well. For example, the Canidae family is represented in 62% of studies on the order Carnivora and 24% of all 115 studies on mammalian hybridization assessed in this systematic review. The second most frequently represented family within Carnivora is the Felidae family, represented in 18% of studies on this order. Canidae and Felidae are the only families within the order Carnivora that include domesticated species, the domestic dog (*Canis lupus familiaris*) and the domestic cat (*F. s. catus)*, respectively. Among the studies included in this systematic review, most (68%) of studies on the Canidae family were focused on hybridization between gray wolves and domestic dogs [38,81,82,83,84]. Accordingly, seven out of eight studies (87%) on the Felidae family were focused on hybridization between domestic cats and wild cats [85,86]. Due to a recent origin of domestic animals, their hybrids with the wild relatives are fertile and thus can back-cross into parental populations [87,88,89]. Moreover, global populations of domestic dogs and cats have been increasing with human population growth, and the majority of individuals globally are free-ranging and thus can breed freely [90]. Widespread occurrence of free-ranging domestic dogs and cats may have promoted their interactions with their wild relatives and as a result increased the rate of hybridization between them [81,91,92]. The presence of a domesticated species within a particular family and order may be thus an important factor increasing the hybridization rate.

Within Artiodactyla, Cervidae, Bovidae and Suidae were the most studied families. All hybridization cases described in the family Suidae were between the wild boar (*Sus scrofa*) and domestic pig (*Sus scrofa domesticus*). Although free-roaming domestic pigs are rare, hybridization with wild boars may occur in open domestic boar farms [93]. Cross-breeding with wild boars is also used intentionally by humans to obtain less aggressive and larger-sized animals, and to increase growth rate of offspring [93]. Climate change, low frequency of predators, supplementary feeding, reforestation of agricultural areas and intentional releases for hunting have led to the range expansion of the wild boar, which as a result has become one of the most widespread large mammals in the world and the second most frequent ungulate in Europe [94,95,96,97]. In many regions, the wild boar has been considered as a pest species for croplands [93,98]. One hypothesis for the vast distribution of wild boars is that introgression from domestic pigs could have led to their increased fitness and invasiveness [99,100]. Hybridization between domestic yaks (*Bos grunniens*) and wild yaks (*Bos mutus*) [101] (Bovidae) is spatially more restricted, given geographically restricted ranges of the wild species, but similarly as in the case of pig–wild boar hybridization, it occurs as both a spontaneous admixture and intentional cross-breeding by humans. Overall, 36% of studies (41 papers) considered in this review were focused on hybridization between domestic animals and their wild relatives, suggesting that the presence of domesticated forms within a family facilitates hybridization.

The effect of human activities on hybridization has long been known [102], and domestication is one of many anthropogenic factors that may increase the frequency of hybridization. The introduction of invasive species to distribution ranges of closely related species may have a similar effect [36,103]. Together with habitat fragmentation and destruction, introduced species are an important threat to global biodiversity [104,105,106,107]. Many wild ungulates are valuable game species, and therefore are strongly affected by humans by extensive translocations and introductions of non-native species, hunting, and artificial management; all these factors contribute to hybridization within ungulate families [108].

In particular, the Cervidae family includes multiple valuable game species. One of them, the sika deer (*Cervus nippon*), was deliberately introduced to many European countries for hunting [109], which has led to hybridization with native deer species in some regions [110,111,112]. Another cervid, European roe deer (*Capreolus capreolus*), is known to hybridize with Siberian roe deer (*Capreolus pygargus*) [15,113] and Italian roe deer (*Capreolus c. italicus*) [114]. Although natural processes (e.g., range expansion) could have caused hybridization in this genus, human-mediated introductions of Siberian roe deer, aimed at increasing body mass and trophy size of European roe deer, affected the rate of hybridization between these species [15]. In Bovidae family, hybridization was reported between Tatra chamois (*Rupicapra rupicapra tatrica*) and introduced Alpine chamois (*Rupicapra rupicapra rupicapra*) [21]. In that case, the introduction was carried out for conservation purposes.

In cetacean species, hybridization has been documented both in captive breeding sites and in the wild [115,116], with around 20% of species known to hybridize [117]. Cross-breeding was shown to be more common between species that have similar morphological and behavioral traits [117,118,119], and to be facilitated by population fragmentation [120,121,122]. Although until recently it was believed that hybridization in cetaceans is a dead-end process, as most known hybrids seemed to be infertile [120], a study on hybridization between fin whale (*Balaenoptera physalus*) and blue whale (*Balaenoptera musculus*) showed that the hybrid individuals can reproduce and survive to adulthood in specific circumstances [123].

### 4.2. Typical Outcomes of Hybridization between Mammalian Species

Depending on species and environmental conditions, hybridization may have either negative or positive impacts, and sometimes there may be very limited consequences. In some cases, hybridization can drive species toward extinction, while in others it provides an opportunity to create new species [124]. Genetic swamping, outbreeding depression, introgression of variants originating from domesticated lineages, and morphological anomalies are typically associated with negative consequences, such as loss of adaptive variation [26,125,126,127,128,129,130], high mortality rates [131], and even extinction [30]. However, hybridization can be considered as a beneficial process in some circumstances. Introgression from a closely related species may facilitate adaptation by providing novel adaptive variation; this may be particularly important when a population occupies a sub-optimal, poor-quality habitat, expands to a new habitat, or experiences rapid changes in local environmental conditions [15,20,132].

**Figure 3:**
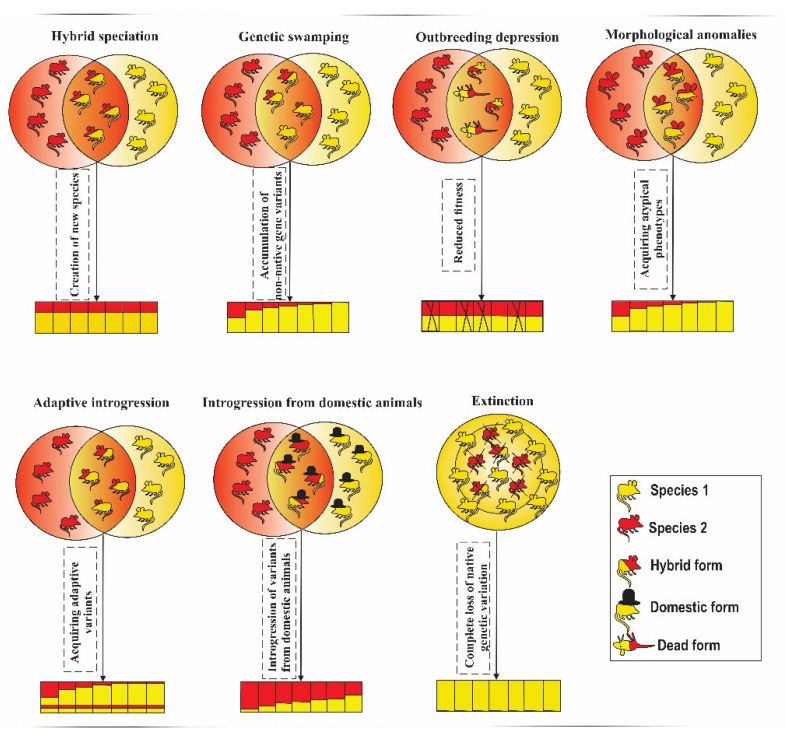
Graphical representation of the common outcomes of hybridization.

Of 13 categories of hybridization outcomes identified in the studies considered in this review (Table 1), genetic swamping and introgression of variants originating from domesticated lineages were two most common outcomes (21% and 18% of studies, respectively). Both these outcomes are commonly considered as negative. Another common outcome (17% of studies), “no or rare evidence of hybridization”, can be classified as neutral. The most common positive outcome (8%) was the gain of novel adaptive variation. Graphical representation of the common outcomes of hybridization is presented on Figure 3.

The frequencies of different outcomes of hybridization may be affected by the specific sets of keywords that were employed in the literature search. We included keywords such as “Genetic swamping”, “Hybrid zone”, “Hybrid speciation”, “Extinction”, and “Outbreeding depression”, and therefore studies focusing on these topics may be overrepresented. Furthermore, information about the consequences of hybridization is missing from some studies, which could also affect the result. Nonetheless, we also identified outcomes that were not included in the keywords, and some which were included had very low frequencies among the selected papers (e.g., “Hybrid speciation”).

#### 4.2.1. Negative Outcomes

##### Genetic Swamping

Genetic swamping refers to the process where genotypes of one or both parental species are partially replaced by hybrid genotypes [32]. Genetic swamping is typically considered as a negative consequence of hybridization due to its disruptive effects on genetic integrity of species and potential to eliminate unique adaptations. Negative results of this process are well documented in some cases, e.g., when it leads to extinction (see below) or results in outbreeding depression e.g., [133]. However, many studies reporting genetic swamping do not assess its fitness consequences or long-term effects on the gene pool composition, and therefore it remains unclear whether the negative consequences of this process prevail among all the cases when it occurs. Genetic rescue, i.e., a reduction of extinction probability of a small, isolated population by restoring gene flow [134] is necessarily associated with genetic swamping, especially if the source of gene flow belongs to another species. Therefore, in some cases negative effects of genetic swamping on the species genetic integrity may be compensated by positive effects, such as reduction of inbreeding depression in isolated populations.

Nearly half (48%) of studies included in this systematic review that reported genetic swamping were focused on hybridization between domesticated mammals and their wild relatives, including wolf and domestic dog [38,81,135], wild boar and domestic pig [20,30,93] and wild cat and domestic cat [44]. More than a quarter (28%) of the studies reported genetic swamping of a native gene pool as the main result of hybridization between introduced species and native species, e.g., in Cervidae [36,136] and Mustelidae families [29,137,138]. Over 80% of the studies reporting genetic swamping focused on cases where hybridization was directly or indirectly caused by human actions, i.e., either domestication or species translocation (deliberate or unintentional). This implies that either such cases are considered as greater concern than introgression resulting from natural hybridization between pairs of wild native species and thus are studied more frequently, or genetic swamping is indeed more frequent when it involves a domesticated or introduced species cross-breeding with a native wild species.

Reproductive barriers between closely related species that evolved in geographic isolation may be weak, and therefore after the secondary contact due to translocation, cross-breeding and production of fertile offspring may be common. In such cases, continuous cross-breeding across generations may result in considerable genetic swamping [36]. Accordingly, reproductive isolation between domesticated mammals and their wild ancestors is frequently incomplete due to their recent divergence. For example, hybridization between wolves and domestic dogs results in introgression of hybridization-derived variants into gene pools of both canids [139]. In wolves, introgression of dog variants is mostly driven by drift, with only a small number of genes experiencing negative or positive selection due to this process [139]. In free-ranging domestic dogs, the observed proportion of candidate genes under positive selection was larger than those under negative selection, suggesting that introgression from wolves may provide dogs with an adaptive advantage [139]. This last case demonstrates that genetic swamping is not an unequivocally negative process, and its outcomes should be considered on an individual basis.

##### Extinction via Genetic Swamping

In extreme cases, extensive genetic swamping may lead a population or an entire species towards extinction [32,140]. Endemic species with patchy, isolated habitats are under a particularly high risk of extinction via hybridization with introduced or invasive closely related species. Of 115 papers considered in this systematic review, three papers mentioned the risk of extinction by genetic swamping [30,114,141]. One of these papers describes the case of the Java warty pig (*Sus verrucosus*), which is endangered with extinction via genetic swamping from the common Indonesian banded pig (*Sus scrofa vitattus*), because of high hybridization rates resulting from the breakdown of reproductive barriers and reduced fertility of hybrids [30]. The second paper reports the case of extinction of the endemic Italian roe deer (*C. c. italicus*) due to extreme genetic swamping from the introduced European roe deer (*Capreolus c. capreolus*) [114]. The third study presents a mathematical model on hybridization between the mountain hare (*Lepus timidus*) and European hare (*Lepus europaeus*), showing that under climate change scenarios, increased hybridization rate can lead to the mountain hare’s extinction via genetic swamping [141]. These studies focused on endemic species, which are threatened by habitat fragmentation and overhunting, and are interbreeding with common, closely related species. Extinction of endemic species through genetic swamping has also been reported in other taxonomic groups, including plants e.g., [140].

Notably, identifying key factors involved in the extinction process is very challenging. In many cases, interaction of different forces such as environmental stress, low genetic diversity and small population size, may lead to the extinction vortex [142,143]. Therefore, while hybridization may play an important role in pushing a species towards extinction, it may not be the only contributing factor. Overall, hybridization contributed to extinction in only 11 documented cases [23].

##### Outbreeding Depression

Outbreeding depression occurs when cross-breeding between two species or populations that are adapted to different environmental conditions results in a loss of local adaptations and reduction of fitness in hybrid individuals [144]. In the studies reviewed here, outbreeding depression has been reported in native species that cross-bred with non-native or invasive species, such as the roe deer interbreeding with the introduced sika deer [36,145], and also in cases of admixture between wild and captive-bred populations e.g., wild versus captive-born American mink, [26]. In both cases, introgression may break-up co-adapted gene complexes, reducing fitness in wild populations and resulting in outbreeding depression [146,147]. Selection pressures in captive-bred populations, associated with adaptations to the captive environment and artificial selection on traits desirable for humans result in the presence of gene variants that are maladaptive in natural habitats [32,148]. Moreover, captive-bred populations have small sizes, which leads to low genetic diversity and inbreeding depression. Introgressive hybridization may introduce maladaptive gene variants present in such captive populations to natural populations, with negative effects on their fitness.

On the other hand, captive breeding programs for conservation purpose have become a conservation tool to prevent extinctions and support reintroductions in cases when remaining wild populations are small and have low genetic diversity [149,150]. For example, captive breeding program of European mink was lunched as a conservation tool for this critically endangered species [151,152].

Among the papers discussed in this systematic review, five studies were focused on admixture between wild and captive-bred populations of the same species. Two of these studies did not detect any signatures of hybridization, and the remaining three studies reported both outbreeding depression and genetic swamping. One of them reported genetic swamping for the native population and increase in genetic diversity for the captive-bred population studied.

##### Introgression from a Domesticated Lineage

Hybridization between wild species and their domesticated relatives frequently results in the introgression of gene variants typical of domesticated animals to wild populations. Although there is a considerable overlap between the studies included in this category with other categories, we consider it separately due to specific conservation problems resulting from this type of introgression [153]. Domesticated mammals are not separated from their wild ancestors by strong reproductive barriers, and therefore they are likely to cross-breed in regions where their ranges overlap. Introgression of domesticated species’ alleles to wild species’ gene pool may threaten the genetic integrity of wild species [154], and therefore it is typically considered as a negative process. However, such introgression may increase the genetic variability in wild species suffering the effects of a severe bottleneck, and/or accelerate the process of adaptation to changing environmental conditions by providing novel genetic variation [155]. For example, the Alpine ibex (*Capra ibex ibex*) acquired one of its two MHC DRB alleles from domestic goats (*Capra aegagrus hircus*), which critically increased diversity of this genetically impoverished species at the key component of the immune system [156].

Among 25 studies from our systematic review that fit in this class, 15 studies focused on hybridization between the grey wolf or the dingo and the domestic dog, four studies focused on the wild boar and the domestic pig, four studies on the wild cat and the domestic cat, one study on the wild sheep and the domestic sheep, and one study on the wild yak and the domestic yak. These studies show that the introgression from domestic animals into their wild relatives is more frequent than in the opposite direction. Population sizes of domestic animals are dependent on the human population size, and therefore human population growth combined with the fragmentation of natural habitats increases both the numbers of domestic animals and the probability of encounters with their wild relatives. Given that wild populations are typically considerably smaller than populations of domestic animals, a single hybridization and back-crossing event will have a larger effect on gene pools of wild populations compared with domestic ones.

Studies focused on hybridization between domestic animals and their wild relatives constituted 36% of studies considered in this systematic review, and thus they had significant impact on the overall proportions of different consequences of hybridization. By default, they were responsible for all the cases of introgression from domesticated lineages, which were reported in 44% of studies focused on hybridization with domestic animals. Further 24.5% of studies reported genetic swamping; in this case, the difference between these two consequences is only in wording, with the exception of introgression cases from wild to domestic populations. The most common positive consequence was gaining novel adaptive variation (7%). Overall, the frequency of hybridization outcomes considered as negative (70%) was considerably higher than the frequency of positive outcomes (9%). This suggests that studies on hybridization between domestic animals and their wild relatives have a disproportional contribution to the negative hybridization outcomes in the overall assessment. However, this is based on the assumption that introgression from domesticated lineages and genetic swamping are negative outcomes by default, which has rarely been tested. Given low divergence between domesticated animals and their wild relatives, it may be expected instead that introgression will rarely lead to increased mortality and infertility of admixed individuals, but the presence of atypical phenotypic traits may result in reduced fitness.

##### Morphological Anomalies

Interspecific hybridization influences phenotypic traits and may create novel or unusual traits [157,158]. Morphological anomalies and abnormal growth are common among hybrid individuals [159] and are sometimes used as a proxy to detect hybridization [160]. Morphological anomalies usually reduce fitness and in extreme cases may cause inviability, and therefore we classified them as a negative outcome of hybridization. In cases where morphological anomalies increase fitness of hybrid individuals, they are considered as novel adaptive variation, which is a separate category of hybridization outcomes identified in this review (see below).

The divergence of phenotypic traits of admixed individuals from average traits within each of the cross-breeding species increases with their divergence time [157,161,162]. Depending on the species, the anomalies can occur in different body parts, including teeth, skull, horn shape, body size etc. Among the papers considered in this systematic review, we found only two papers that reported morphological anomalies in hybrid individuals, including abnormal placental growth in hybrids between two species of dwarf hamsters (*Phodopus campbelli* and *Phodopus sungorus*) [131] and skull, dental and horn anomalies in the wildebeest hybrids (*Connochaetes taurinus* and *Connochaetes gnu*) [157].

##### Loss of Reproductive Output

Hybridization can change reproductive output by leading to changes in mating behavior [163] or by wasting reproductive efforts. These changes typically involve reduction in reproductive success, and therefore are considered as a negative consequence of hybridization. For instance, unidirectional introgression from the fin whale to blue whale resulted in the reduction of reproductive rate of the blue whale, reducing its recovery [123]. In cases when most hybrid individuals are infertile and inviable, introgression does not happen or is rare, and therefore the consequences of hybridization are reduced to the production of F1 hybrids only. Moreover, in some cases the reproductive output differs between different generations of hybrids. For example, hybridization between *Microtus hartingi lydius* and *Microtus hartingi strandzensis* produces viable and prolific F1 hybrids, while in the F2 generation, males are sterile and the mortality rate is high [164]. Falling fertility rates and loss of reproductive outputs may lead to severe demographic declines in parental species and even a rapid extinction of local populations involved in cross-breeding.

#### 4.2.2. Positive Outcomes

##### Increase in Genetic Diversity and Reduction of Inbreeding

In a specific case when genetic diversity of a population is very low and the rate of inbreeding is high, introgression from a non-native population or species can increase genetic diversity without any signs of outbreeding depression. This can be considered as a positive consequence of hybridization. Moreover, in small and fragmented populations that have low genetic diversity and experience inbreeding depression, hybridization can restore population viability [165,166]. Genetic rescue, i.e., restoration of genetic diversity and mitigation of inbreeding depression through gene flow can be a valuable tool in conservation of small, isolated populations [167]. For instance, introgressive hybridization with a non-native Alpine chamois (*R. r. rupicapra*) was shown to improve genetic diversity of Tatra chamois (*R. r. tatrica*), an endangered endemic population in the Tatra Mountains that suffered from a high level of inbreeding depression [21].

Although introgression from domesticated lineages can be considered as a threat for wild populations, in some circumstances it can increase genetic diversity and viability of wild populations. For example, increase in genetic diversity has been reported in European wild boars that cross-bred with domestic pigs [20]. Accordingly, admixture between feral and farmed populations of American mink (*Neovison vison*) increased genetic diversity of the invasive populations of these species in Europe, which could increase their adaptive potential and therefore compromise management efforts to control them [137]. Although this is a negative process from the conservation perspective, it can be considered as a positive outcome of hybridization in terms of increasing individual fitness in the invasive population.

##### Novel Adaptive Variation

In some cases, creation of novel genetic diversity via hybridization can facilitate species adaptation to variable or novel environmental conditions, without a loss of its genetic integrity [132,168]. Admixed individuals may acquire new adaptive traits, providing them with selective advantages in comparison to their parental species [169,170]. For instance, in eastern Poland, introgression from the Siberian roe deer (*C. pygargus*) allowed the European roe deer (*C. capreolus*) to adapt better to severe winters, which are an important contributing factor of roe deer mortality [171]. Furthermore, hybridization between the coyote (*Canis latrans*) and the grey wolf (*Canis lupus*) in Canada has resulted in the introduction of novel adaptive variation to the coyote populations, allowing them to increase in body size, which in turn improved their success in hunting deer [172].

In several mammalian species, including humans, the presence of adaptive variation from their extinct relatives has been detected [173,174,175], implying that ancient hybridization events provided long-lasting positive fitness effects [176]. For example, Tibetan and Himalayan wolves experienced ancient introgression from an unknown canid lineage, which resulted in the introgression of an *EPAS1* haplotype that confers an adaptive advantage in high altitude environments [175]. In humans, ancient cross-breeding with Neanderthals and Denisovans in Eurasia resulted in introgression of novel adaptive variation, but also increased the genetic load compared with non-admixed African populations [25,177]. Altogether, among 14 papers considered in this systematic review that used genome-wide SNPs or whole genome sequencing, there were six papers reporting cases of ancient introgression. Three of these studies showed that ancient introgression was associated with gaining novel adaptive variation, and the remaining three papers reported ancient introgression without investigating its consequences.

##### Hybrid Speciation

Hybrid speciation refers to the process in which hybridization results in the creation of a new species, which is characterized by mixed ancestry and distinct genetic composition from its parental species [18]. Hybridization may act as a driving force in speciation by creating new hybrid phenotypes or providing necessary material for adaptive divergence [17]. Given that the creation of a new species increases biodiversity, it can be considered as a positive outcome of hybridization.

Three criteria should be met to demonstrate speciation via hybridization; first, confirmed evidence of a past hybridization event in the putative hybrid species, second, reproductive isolation between the hybrid species and its parental species, and finally the presence of isolating impacts of hybridization [178]. Hybrid speciation may allow the new species to colonize a new habitat [179].

In this review we found four studies that reported hybrid speciation [131,180,181,182]. These studies showed that the emergence of distinct phenotypic traits in hybrid individuals may play an important role in speciation by impeding gene flow between parental species and hybrid individuals [131]. For instance, differentiation in facial patterns in the primate genus *Cercopithecus* is one of the key mechanisms driving hybrid speciation in this genus [181,183]. Abnormal growth patterns in hybrids between two dwarf hamster species, *P. campbelli* and *P. sungorus*, were suggested to play an important role in speciation by contributing to reproductive isolation between these recently diverged species [131]. Despite its potentially important role in mammalian speciation, the genetic basis of growth-related developmental inviability is still unknown [131]. However, studies on a hybrid zone between subspecies the house mouse (*Mus musculus*), which is a model species in genetics, provided an insight into the molecular mechanisms underlying hybrid speciation. Dysfunction in the Mecp2 protein in the house mouse resulting from introgressive hybridization within the hybrid zone may induce changes in the expression of thousands of genes, which may initiate the speciation process [182].

#### 4.2.3. Neutral or Unspecified Outcomes

##### Intermediate Phenotypic Traits

Although in some cases interspecific hybridization may create deleterious morphological anomalies or novel adaptive traits (see above), hybrid individuals frequently show intermediate phenotypic traits compared to their parents [184]. The additive effect, dominance effect, and/or epistatic effect may create variation in polygenic traits [157,185]. In the additive model, F1 hybrid offspring shows intermediate phenotypes relative to their parents [185]. A classic example of the effect of hybridization on morphological traits are the Darwin finches in Galapagos, where most hybrid individuals have intermediate body size and beak shape compared with the parental species [186]. Several studies in this review reported intermediate phenotypes, e.g., in cetaceans [187], mustelids [188], camelids [189] and primates [181]. The presence of admixed individuals with intermediate phenotypes may impede species identification in the field. For instance, field identification of four chipmunk species (*Tamias* spp.) in the Sierra Nevada, USA, was associated with 14% error rate, which was in part attributed to sporadic hybridization among these species [190]. The fitness consequences of intermediate phenotypic traits have rarely been studied and therefore this outcome of hybridization could not be classified as either positive or negative.

##### Hybrid Zones

Hybrid zones are areas where two genetically distinct linages meet, mate and create viable offspring [191]. These geographic regions are usually narrow, with the width ranging from several meters to several kilometers [192]. Hybrid zones can be created through natural hybridization between parapatric or sympatric species [193,194]. Most hybrid zones are maintained by the balance between natural selection against hybrids and dispersal capabilities of the cross-breeding taxa [179,191]. If before the range expansion or removal of a geographic barrier, reproductive isolation between closely related species has not been complete, hybrid zone may be formed. For example, due to a recent divergence and weak reproductive isolation between the pine marten *Martes martes* and the sable *Martes zibellina* in Western Siberia, a vast hybrid zone has formed between these species after the Last Glacial Maximum [138]. Features of hybrid zones, such as fertility or sterility of hybrid individuals, directionality of mating, hybridization frequency, and geographic extent of introgression, vary considerably, and their examination can help understand the mechanisms of hybrid zone maintenance [195]. In woodrat species (*Neotoma bryanti* and *Neotoma lepida*), a hybrid zone has been maintained as a result of sporadic cross-breeding between these species and hybrid fertility [196]. Among studies included in this literature review, hybrid zones have been described in mice (*Mus musculus musculus* and *Mus musculus domesticus*) [197], different woodrat species [195,196,198,199,200], marmots [201], primates [181], artiodactyles [157,202], Diprotodontia [203,204] and carnivores [138,205].

##### Hybridization without Significant Impacts

Some studies included in this review showed that despite hybridization, populations maintained their genetic distinctiveness [206]. For instance, despite extensive rate of hybridization among different bat species in Poland, their gene pools have not been disrupted by introgression [74]. Furthermore, admixture between Italian wolves and domestic dogs did not affect the integrity of wolves’ gene pool [207]. The lack of significant effects on the gene pool does not necessarily imply the lack of any effects, e.g., loss of reproductive effort. Given that these effects were not studied, we classified this type of outcome as neutral.

##### No or Rare Evidence of Hybridization

This category includes studies that have not found any signs of hybridization in the populations studied or found only very limited evidence e.g., [154,208,209,210,211,212,213,214,215]. We found 24 studies that fitted this category. This could include cases where hybridization was rare or did not occur, as well as cases where limitations in sampling and the use of low number of genetic makers could result in poor detection of hybridization [216,217,218,219,220,221,222,223,224,225]. The number of genetic markers is important to detect signatures of hybridization, especially if cross-breeding and/or back-crossing events are rare [56,124]. Moreover, small data sets may show only a preliminary assessment of hybridization [226], and comprehensive sampling is necessary to obtain reliable results.

In some cases, efficient conservation management may result in low rate of hybridization [212,227]. For example, because of careful monitoring and management, the Scandinavian wolf population shows a lower level of hybridization with dogs compared to other European wolf populations, which was demonstrated based on the comprehensive sampling and the analysis of whole genomes [82]. The rate of hybridization between the same pairs of species may differ regionally depending on environmental conditions [228]. For example, hybridization between bat species *Myotis myotis* and *Myotis blythii* has been reported in Europe, but in Turkey no signs of hybridization between these species have been detected [229].

Altogether, 38% of the studies assessed in this systematic review reported neutral or unspecified outcomes of hybridization. Within this group, “no or rare evidence of hybridization” was the most common hybridization outcome, which occurred in 17% of all the studies on hybridization. This result suggests that one of the five categories of hybridization outcomes delineated for the purpose of species conservation [35], “negligible impact and minimal introgression of genes into the species of concern”, occurs relatively frequently. Therefore, a presumption that hybridization always constitutes a threat to biodiversity is incorrect, and instead the decision-making regarding the management and conservation of wild-living hybrids should be based on the examination of hybridization outcomes case-by-case [35].

#### 4.2.4. Consequences of Hybridization for Threatened Species

Only 18% of studies considered in this review were focused on threatened species (having the IUCN Red List categories of Near Threatened (NT), Vulnerable (VU), Endangered (EN), Critically Endangered (CR), Extinct in the Wild (EW)). Negative consequences (e.g., genetic swamping, extinction via genetic swamping, introgression from domesticated lineages and loss of reproductive output) were reported in 38% of these studies and positive consequences (with only one category, gaining novel adaptive variation) were reported in 14% of them. In the remaining studies, the authors did not mention any positive or negative consequences or did not find any evidence of hybridization. The frequency of the positive consequences reported for all the studies assessed in this systematic review was very similar (13%), while the frequency of the negative consequences was higher (49%) compared to those observed in the studies on threatened species. This suggests that the negative consequences of hybridization are not intensified in endangered species, at least the mammalian species considered in this review.

## 5. Conclusions

Among the papers included in this systematic review, hybridization outcomes typically considered as negative had considerably higher frequency (49%) than those considered as positive (13%). However, these frequencies could have been biased by several factors and therefore should be treated with caution. For instance, two most frequent outcomes of hybridization, genetic swamping and introgression of variants from domestic animals are typically considered as negative, but this is not always the case. In some circumstances, moderate levels of genetic swamping or introgression of domestication-related variants may result in increased fitness and genetic rescue [155,230]. The cases where hybridization outcomes are unequivocally negative, leading to extinction or loss of reproductive output, are relatively rare. They were reported in 13% of the studies considered in this review—the same frequency as that of the positive outcomes. In cases when the hybridization outcome cannot be easily determined, e.g., when genetic swamping occurs at a low rate, long-term monitoring of admixed populations is required to conclude about advantages and disadvantages of introgressive hybridization. For this purpose, at least two consecutive generations should be monitored [12,13], but currently, many studies are based on a singular sampling effort, and fitness of sampled individuals is rarely assessed.

It is also important to stress that the detection of different outcomes of hybridization depends on the type of molecular markers applied. Microsatellite markers enable identification of first-generation hybrids and recent back-crosses, but cannot reliably detect more distant hybridization events [56]. Since microsatellites are neutral genetic markers and are typically genotyped in small numbers (<100), they cannot be used to detect adaptive introgression or hybrid speciation, which are among the most frequently reported positive outcomes of hybridization. In contrast, some negative outcomes, such as genetic swamping, can be detected using a small number of neutral markers. Given that until recently microsatellite loci were the most frequently chosen markers in hybridization studies, and they were used in 50% of studies considered in this systematic review, the frequency of negative outcomes of hybridization may be overestimated.

A combination of neutral loci and those located within coding genes is better suited to provide an unbiased insight into the relative frequencies of positive and negative hybridization outcomes and identify factors that affect them. Single Nucleotide Polymorphisms (SNPs) can be genotyped in large numbers (hundreds of thousands to millions) using arrays or next generation sequencing, which makes them suitable for identification of adaptive loci [231,232] as well as detection of small proportions of hybrid ancestry and identification of F2-F4 backcrosses [233,234]. Therefore, the application of this type of genetic markers creates an opportunity to identify both positive and negative impacts of hybridization.

In some circumstances, hybridization can be used as a conservation tool to facilitate adaptation of populations to changing habitat conditions and to increase individual fitness in populations experiencing inbreeding depression [12,13]. To use hybridization this way, we need to achieve a better understanding on how to prevent negative effects of hybridization without eliminating the potential for the positive effects. This will require comprehensive studies focusing on the genetic effects of hybridization on both neutral and functional parts of the genome and fitness effects of cross-breeding on F1 hybrids and several generations of back-crosses. Experimental studies simulating different evolutionary scenarios may be the best way to achieve an unbiased assessment of the frequency of different hybridization outcomes.

## Figures and Tables

**Table 1:** Outcomes of hybridization described in papers included in the systematic review. The outcomes are grouped by the character of their impact. The reported frequencies of different hybridization outcomes in the papers studied should not be considered as reliable estimates of the real frequencies.

	Results	Impacts	Number of Papers	Percentage	Description
1	Genetic swamping	Considered as negative	29	20.71	Genetic integrity of a species involved in hybridization being threatened by introgression from another species
2	Introgression from a domesticated lineage	Considered as negative	25	17.85	Genetic integrity of wild species being threatened by introgression from a domesticated lineage
3	Extinction due to extreme genetic swamping	Negative	3	2.14	Complete loss of genetic material of one of the species involved in hybridization
4	Outbreeding depression	Negative	7	5.0	Reduction or loss of specific adaptations and overall fitness
5	Morphological anomalies	Negative	2	1.4	Morphological anomalies with negative effects on fitness
6	Loss of reproductive output	Negative	3	2.14	Decrease in growth rate of parental species because of wasted reproductive effort
7	Increase in genetic diversity and reduction of inbreeding	Positive	3	2.14	Increase in genetic diversity via low rates of introgression, without any evidence of outbreeding depression; reduction of inbreeding levels
8	Gaining novel adaptive variation	Positive	11	7.85	Transferring of adaptive variants through hybridization
9	Hybrid speciation	Positive	4	2.85	Creation of a new species via hybridization
10	Intermediate phenotypic traits	Neutral or unknown	10	7.14	Intermediate morphological characteristics of hybrid individuals relative to the parental species
11	Hybrid zone	Neutral or unknown	14	10.0	Geographically restricted zones where genetically distinct species meet and mate
12	Hybridization without significant impacts	Neutral or unknown	5	3.57	Evidence of hybridization without substantial changes in the gene pools of each species
13	No or rare evidence of hybridization	Neutral or unknown	24	17.14	Hybridization is rare and does not result in introgression

## Data Availability

The references of 115 papers used as data source in the systematic review are provided in the reference list. In addition, the list of these 115 papers is provided in the Supplementary Table S1. The summary data from these studies is provided in the Results section.

## Supplementary Materials

The following are available online at https://www.mdpi.com/article/10.3390/genes13010050/s1, Table S1: A list of papers considered in the systematic review.
